# Enrichment of provitamin A content in wheat (*Triticum aestivum* L.) by introduction of the bacterial carotenoid biosynthetic genes *CrtB* and *CrtI*

**DOI:** 10.1093/jxb/eru138

**Published:** 2014-04-01

**Authors:** Cheng Wang, Jian Zeng, Yin Li, Wei Hu, Ling Chen, Yingjie Miao, Pengyi Deng, Cuihong Yuan, Cheng Ma, Xi Chen, Mingli Zang, Qiong Wang, Kexiu Li, Junli Chang, Yuesheng Wang, Guangxiao Yang, Guangyuan He

**Affiliations:** The Genetic Engineering International Cooperation Base of Chinese Ministry of Science and Technology, The Key Laboratory of Molecular Biophysics of Chinese Ministry of Education, College of Life Science and Technology, Huazhong University of Science and Technology, Wuhan, China

**Keywords:** Bacterial phytoene synthase (CrtB), bacterial phytoene desaturase (CrtI), carotenoid β-hydroxylase (HYD), lycopene β-cyclase (LCYB), provitamin A, particle bombardment, transgenic wheat.

## Abstract

Carotenoid content is a primary determinant of wheat nutritional value and affects its end-use quality. Wheat grains contain very low carotenoid levels and trace amounts of provitamin A content. In order to enrich the carotenoid content in wheat grains, the bacterial phytoene synthase gene (*CrtB*) and carotene desaturase gene (*CrtI*) were transformed into the common wheat cultivar Bobwhite. Expression of *CrtB* or *CrtI* alone slightly increased the carotenoid content in the grains of transgenic wheat, while co-expression of both genes resulted in a darker red/yellow grain phenotype, accompanied by a total carotenoid content increase of approximately 8-fold achieving 4.76 μg g^–1^ of seed dry weight, a β-carotene increase of 65-fold to 3.21 μg g^–1^ of seed dry weight, and a provitamin A content (sum of α-carotene, β-carotene, and β-cryptoxanthin) increase of 76-fold to 3.82 μg g^–1^ of seed dry weight. The high provitamin A content in the transgenic wheat was stably inherited over four generations. Quantitative PCR analysis revealed that enhancement of provitamin A content in transgenic wheat was also a result of the highly coordinated regulation of endogenous carotenoid biosynthetic genes, suggesting a metabolic feedback regulation in the wheat carotenoid biosynthetic pathway. These transgenic wheat lines are not only valuable for breeding wheat varieties with nutritional benefits for human health but also for understanding the mechanism regulating carotenoid biosynthesis in wheat endosperm.

## Summary

Co-expression of *CrtB* and *CrtI* enhanced carotenoid in endosperm through upregulation of the endogenous carotenogenic genes. Our results also indicate important roles of *LCYB* and *HYD* in wheat carotenoid biosynthesis.

## Introduction

Carotenoids are one of the most diverse classes of natural pigments produced by plants, algae, fungi, and bacteria, and have multiple functions because of their colour characteristics and antioxidant activities. In plants, they guarantee the photosynthesis process by correcting assembly of the photosystems and scavenging reactive oxygen species derived from excess light energy. They confer their colour in plant organs to attract pollinators and seed dispersers for plant reproduction (Demmig-Adams and Adams 1996; DellaPenna and Pogson, 2006). Dietary carotenoids are essential for humans because mammals are incapable of *de novo* synthesis of vitamin A. Consumption of carotenoids in the human diet is thus beneficial to health. There is abundant evidence showing that carotenoids are effective in preventing age-related macular degeneration and certain cancers (Rao and Rao, 2007; Singh and Goyal, 2008). Currently, carotenoids are also utilized as a healthcare food, food additives, and cosmetic colorants for their huge commercial value (Sandmann, 2001).

**Fig. 1.**
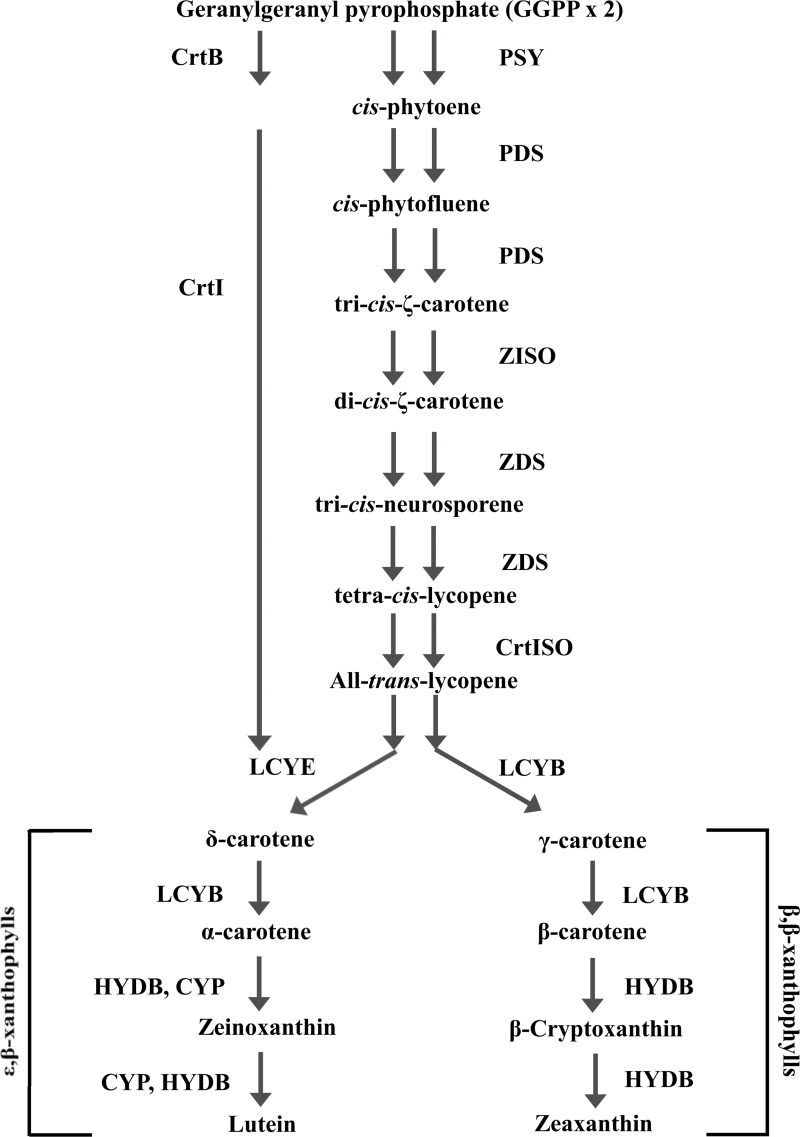
The carotenoid biosynthetic pathway in wheat. PSY, phytoene synthase; PDS, phytoene desaturase; ZDS, ζ-carotene desaturase; ZISO, ζ-carotene isomerase; CrtISO, carotenoid isomerase; LCYB, lycopene β-cyclase; LCYE, lycopene ε-cyclase; HYD, carotenoid β-hydroxylases (non-haem di-iron type); CYP, carotenoid ε-hydroxylase (cytochrome P450 type); CrtB, bacterial homologue of PSY; CrtI, bacterial homologue of PDS, ZDS, and CrtISO.

In higher plants, carotenoid biosynthesis originates in the plastids catalysed by the nuclear-encoded enzymes and they are then imported post-translationally into the organelle (Howitt and Pogson, 2006; Rodríguez-Concepción, 2006). Phytoene synthase (PSY in plants or CrtB in bacteria) catalyses the condensation of two geranylgeranyl diphosphate molecules to produce phytoene. This is the first rate-limiting step in carotenoid biosynthetic pathway. After that, phytoene undergoes four desaturation reactions to form lycopene by the desaturases phytoene desaturase (PDS) and ζ-carotene desaturase (ZDS). The poly-*cis* intermediate carotenoids are converted to their all-*trans* forms by the actions of two carotenoid isomerases, ZISO and CrtISO. The bacterial carotene desaturase, CrtI, is capable of performing all the above-mentioned desaturation and isomerization reactions. A major branch point exists after lycopene synthesis. The cyclization of lycopene mediated by the enzymes lycopene-β-cyclase (LCYB) and lycopene-ε*-*cyclase (LCYE) gives rise to α-carotene and β-carotene. Subsequently, these molecules are hydroxylated by non-haem di-iron carotenoid β-hydroxylase (HYD) and by ε-hydroxylase cytochrome P450 carotene hydroxylases (CYPs). In the β-carotene branch, this yields β-cryptoxanthin, which is further converted into zeaxanthin, whereas in the α-carotene branch the conversion yields lutein (Fig. 1) (reviewed by Giuliano *et al.*, 2008; Cazzonelli and Pogson, 2010; Moise *et al.*, 2014).

As carotenoids have many health-promoting properties, numerous attempts have been made to identify the genes in the carotenoid biosynthetic pathways and to improve the carotenoid content in crops by metabolic engineering. Successful reports in canola, tomato, rice, and maize with enhanced carotenoid content and composition have demonstrated the feasibility of improving the nutritional value of crop species through genetic engineering (reviewed by Farré *et al.*, 2011). Heterologous expression of carotenoid biosynthetic genes and the possible functional divergence of the endogenous carotenoid biosynthetic pathway could result in the production of unintended carotenoids and unexpected effects on the endogenous carotenogenic genes in transgenic plants. Therefore, confirming the application potential of widely used carotenoid biosynthetic genes, such as *CrtI* and *CrtB*, and elucidating the diversified gene functions in the carotenoid biosynthetic pathway among different plant species will facilitate further genetic engineering of carotenoid biofortification (Sandmann *et al.*, 2006).

As one of the ‘big three’ cereal crops, wheat supplies the greatest amount of calories, proteins, and vitamins for humans (Shewry, 2009). Almost 94% of the wheat grown today is bread wheat (*Triticum aestivum* L.), and it is used mainly for making bread and other flour-based food (Li *et al.*, 2014). Carotenoid content is one of the major determinants of wheat nutrient quality (Mares and Campbell, 2001). Wheat grains contain only a small amount of carotenoids and accumulate mainly lutein, which is devoid of provitamin A activity (Hentschel *et al.*, 2002). Our understanding of wheat carotenoid biosynthesis has lagged behind in contrast to other staple crops. The regulation of carotenoid biosynthesis in wheat is poorly understood, although the carotenoid biosynthetic pathway has been characterized in the cereals. The identification of key carotenoid biosynthetic genes has progressed comparative slowly due to the large size and complexity of the wheat genome (hexaploid wheat is 16 Gb; Brenchley *et al.*, 2012). Several wheat carotenoid biosynthetic enzymes have been identified, such as PSY, PDS, ZDS, LCYE, and HYD (Howitt *et al.*, 2009; Wang *et al.*, 2009; Cong *et al.*, 2010; Qin *et al.*, 2012). Meanwhile, the bottlenecks in the genetic transformation have implied that genetic engineering could still not be used routinely for wheat research (Jones, 2005). Based on the above-mentioned reasons, few studies on carotenoid metabolic engineering in wheat have been reported. Our group has previously generated transgenic wheat with enhanced total carotenoid levels by co-transformation with maize *PSY1* and *CrtI* (Cong *et al.*, 2009), but the elevation of the carotenoid content was moderate compared with the donor wheat cultivar EM12, which contains relatively higher levels of lutein. In addition, the profound effects of transgenes on altering the carotenoid compositions and on endogenous carotenoid biosynthetic pathway remained unclear. Therefore, it is of significance not only to improve the carotenoid content in wheat grains but also to obtain a comprehensive understanding of the endogenous carotenoid biosynthetic pathway and metabolic regulation of the pathway in transgenic wheat. In the present study, the bacterial carotenoid biosynthetic genes *CrtB* and *CrtI* were introduced into the low carotenoid wheat cultivar Bobwhite. To our knowledge, this is the first report on the generation of transgenic wheat with high provitamin A accumulation.

## Materials and methods

### Plant materials

Wheat plants (*T. aestivum* L. cv. Bobwhite) were grown in an experimental field at Huazhong University of Science and Technology in Wuhan, China. This wheat variety was chosen because of its low carotenoid content in endosperm. A transgenic wheat line expressing *ZmPSY1* and bacterial *CrtI*, designated OEIP, were used as a control of the transgenic wheat lines present in this study (Cong *et al.*, 2009).

### Plasmid constructs

Plasmids pACCRT-EB and pYPIET4 served as the source of the coding sequences of the *Erwinia uredovora CrtB* and *CrtI* genes, respectively (kindly provided by Dr Sandmann from the Botanisches Institut, FB Biologie, J. W. Goethe Universitat, Germany, and Central Laboratories for Key Technology, Kirin Brewery Co., Japan).

**Fig. 2.**
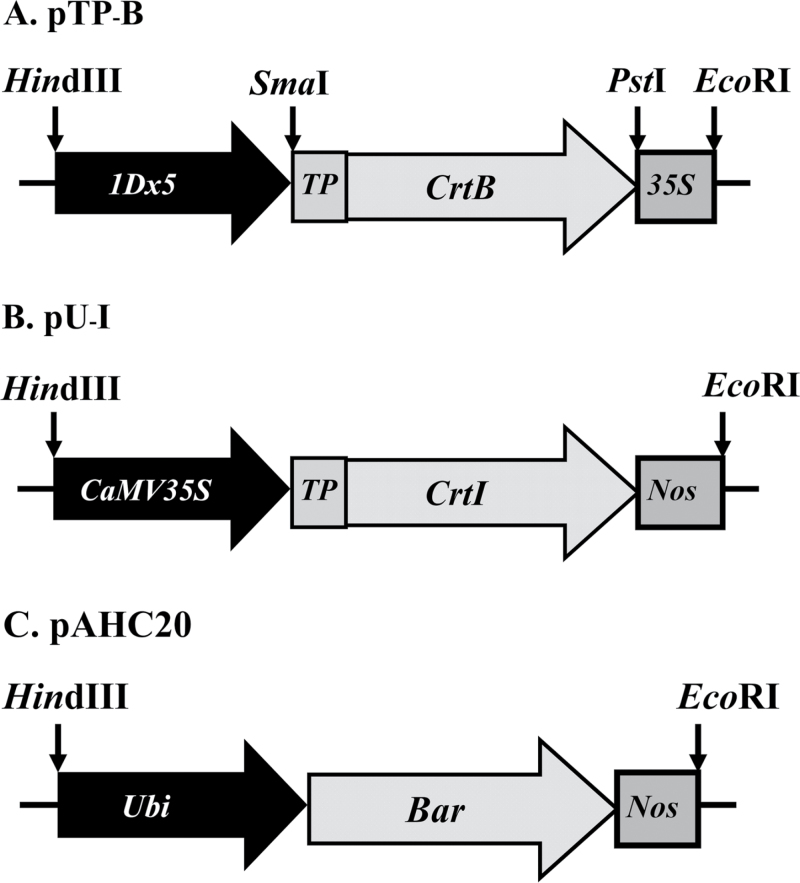
Structures of the three wheat transformation plasmids (pTP-B, pU-I, and pAHC20) used in this study. *1Dx5*, endosperm-specific promoter of the high-molecular mass glutenin subunit gene *1Dx5* from wheat; *CaMV35S*, cauliflower mosaic virus (CaMV) 35S promoter; *Ubi*, constitutive maize *Ubiquitin-1* promoter; *TP*, transit peptide from pea Rubisco small subunit (*rbcS*); *CrtB*, phytoene synthase gene from *E. uredovora*; *CrtI*, carotene desaturase gene from *E. uredovora*; *Bar*, bialaphos resistance gene; *NOS*, *Agrobacterium tumefaciens* nopaline synthase (*NOS*) terminator.

In order to direct the expression of *CrtB* and *CrtI* in wheat plastids, sequences encoding the transit peptide (TP) of the small subunit of Rubisco (*rbcS*) from pea (*Pisum sativum* L.) were fused with the *CrtB* or *CrtI* gene during vector construction (Fig. 2).

To generate pU-I, the *CaMV 35S promoter::TP-CrtI::NOS* cassette was cut from pYPIET4 with *Eco*RI and *Hin*dIII, and the resulting 3.1kb fragment was ligated into the *Eco*RI–*Hin*dIII site of the vector pUC18 (Takara, Dalian, China).

To generate pTP-B, the *CrtB* gene was amplified from pACCRT-EB using primers CrtB-F1 and CrtB-R1. The *TP* gene was amplified from the pYPIET4 using primers TP-F1 and TP-R1. The obtained fragments were then spliced by an overlap-extension PCR using primers *Sma*I-TP-F2 and *Pst*I-CrtB-R2. The resulting *TP-CrtB* fragment was digested with *Sma*I/*Pst*I and ligated into pLRPT under the control of an endosperm-specific *1Dx5* promoter and CaMV 35S terminator to generate plasmid pTP-B (the primers used are listed in Supplementary Table S1 at *JXB* online).

Plasmid pAHC20 carrying a selectable *bar* gene, which confers resistance to the herbicide phosphinothricin, was also used. The pU-I, pTP-B, and pAHC-20 plasmid constructs were sequenced to verify that the manipulations did not introduce errors.

### Wheat transformation and regeneration

Wheat genetic transformation was performed according to the bombardment method reported by Sparks and Jones (2004). Immature scutella (14 d after pollination) from the wheat cultivar Bobwhite were transformed with the plasmids pTP-B:pU-I:pAHC20 at a 2:2:1 molar ratio, pTP-B:pAHC20 at a 3:1 molar ratio, and pU-I:pAHC20 at a 3:1 molar ratio. The regenerated plantlets were selected on medium with 3mg l^–1^ of phosphinotricin. The surviving plants were transferred to soil and grown to maturity under growth chamber conditions of 22/16 °C day/night temperature, 50–70 % relative humidity, 16/8h light/dark cycle, and 300 μmol m^–2^ s^–1^ photosynthetic photon flux density. The regenerated plants were confirmed by PCR amplification using gene-specific primers (Supplementary Table S1 available at *JXB* online). The PCR-positive transgenic plants were self-pollinated, and the non-segregant lines for transgenic *CrtB* and/or *CrtI* were selected for analyses of carotenoid composition and expression levels of carotenoid biosynthetic genes (Supplementary Fig. S1 at *JXB* online).

### Analysis of carotenoid composition by high-performance liquid chromatography (HPLC)

Carotenoids in the mature wheat seeds were extracted according to Liu *et al.* (2007). Seed samples were freeze dried with liquid nitrogen and ground into a fine powder. A 1g aliquot of the lyophilized powder was homogenizer in 15ml of extracting solvent (hexane:acetone:ethanol, 50:25:25, v/v/v) containing 0.01 % (w/v) 2,6-di-*tert*-butyl-methylphenol (BHT, Sigma, Shanghai, China) in an ultrasonic cleaner for 30min (SB-5200DTN, Scientz, China), and centrifuged for 10min at 4000*g* under 4 °C (CR-21G, Himac, Japan). The coloured supernatant was collected and transferred to a 50ml volumetric centrifuge tube, and the residue was re-extracted several times with the same extracting solvent until colourless. The combined supernatant was then washed three times with saturated NaCl solution until neutral, and the aqueous phase was discarded. The supernatant were concentrated to dryness evaporated under N_2_, redissolved in 0.3ml methyl tert-butyl ether containing 0.01 % (w/v) BHT. After centrifuging at 12 000rpm at 4 °C for 30min, the sample was filtered through a 0.22 μm filter before HPLC analysis. For quantitative purpose, β-apo-8′-carotenal was added to each sample as an internal standard prior to extraction (10 μg g^–1^ of freeze-dried sample).

To quantify the carotenoids and chlorophylls in leaf tissues, three mature leaves from the plants of each transgenic and untransformed wheat lines (approximately 4 months after germination) were used for extraction, followed by HPLC analysis according to Fraser *et al.* (2000). The internal standard was also added prior to extraction. To avoid degradation of carotenoids, the extraction experiment (from both seeds and leaves) was performed under dim light, and all samples were kept on ice during the whole process. The carotenoids were extracted from seeds with three replicates, while the extraction of pigments from leaves was performed with four replicates.

Chromatography was carried out with a Waters HPLC system equipped with a model 1525 solvent delivery system, a model 2996 photodiode array detection system and a Breeze2 Chromatography Manager. A C_30_ carotenoid column (150×4.6mm, packing 3 μm) from YMC (Wilmington, NC, USA) was used for separation. Throughout chromatography, the eluate was monitored continuously from 200 to 700nm. The column was operated at 25 °C and eluted with a gradient mobile system consisting of solvent A (acetonitrile:methanol, 3:1, v/v), containing 0.01 % BHT and 0.05 % triethylamine (Sigma-Aldrich, Shanghai, China), and solvent B (100% methyl tert-butyl ether), containing 0.01 % BHT. The gradient was programmed as follows: 0–10 min: A:B, 95:5; 10–19 min: A:B, 86:14; 19–29 min: A:B, 75:25; 29–54 min: A:B, 50:50; 54–66 min: A:B, 26:74, and back to the initial conditions for re-equilibration. All HPLC-grade solvents were purchased from JT Baker (Phillipsburg, USA). Calibration standards for α-carotene, *trans*-β-carotene, β-cryptoxanthin, lutein, β-apo-8′-carotenal, zeaxanthin, *trans*-lycopene, chlorophyll *a*, and chlorophyll *b* were purchased from Sigma-Aldrich; phytoene, 9-*cis*-β-carotene, neoxanthin and violaxanthin were purchased from Carotenature (Lupsingen, Switzerland). These standards, and the β-apo-8′-carotenal internal standard, were used to generate standard calibration curves. Carotenoids and chlorophylls were identified by comparing the retention time and spectra with published data and then quantified from their peak areas (Fraser *et al.*, 2000; Sander *et al.*, 2000; Rodríguez-Amaya and Kimura, 2004; Liu *et al.*, 2007).

### Quantitative PCR (qPCR) analysis

Total RNA extracted from seeds at 25–30 d after pollination and from 4-month-old seedlings of transgenic and untransformed wheat lines with RNAiso Plus (Takara), and then treated with RNase-free DNase I (Fermentas, Shenzhen, China) to remove any residual genomic DNA that might be carried through the extraction process, following the manufacturer’s instructions. RNA concentration and purity (*A*_260_/*A*_280_ ratios) were determined using a Nanodrop^TM^ ND-1000 spectrophotometer (Thermo Scientific, Wilmington, USA). An aliquot of RNA samples was separated on a non-denaturing agarose gel to assess its integrity. Reverse transcription was performed with 1.0 μg of total RNA using a RevertAid^TM^ first -trand cDNA synthesis kit (Fermentas). The primers used for qPCR analysis are listed in Supplementary Table S1 (available at *JXB* online).

Quantitative reverse transcription-PCR (qRT-PCR) analysis was done with the LightCycler II System (Roche Diagnostics, Basel, Switzerland) using LightCycler FastStart SYBR Green Master Mix (Roche Applied Sciences, Indianapolis, USA). Amplification was carried out using the following cycling parameters: 40 cycles of 95 °C for 15 s and 60 °C for 60 s. Fluorescence was acquired at 60 °C. The specificity of the unique amplification product was determined by a melting-curve analysis from 55 to 99 °C. Data were analysed using the Lightcycler software version 4 and normalized to expression of the wheat β-actin gene, as it has relatively constitutive expression levels throughout the wheat developmental process. Negative controls with no template and no reverse transcription were also assayed for each primer pair to verify the quality of the cDNA templates and PCR amplifications. Dissociation-curve analysis was performed following qPCR and a single peak was observed for each primer pair. A portion of the qPCR products was separated on agarose gels and single products of the expected sizes were detected.

### Southern blotting analysis for the non-segregant transgenic lines

Total genomic DNA was extracted from leaves of T_3_ transgenic plants using a cetyltrimethyl ammonium bromide extraction method (Sambrook *et al.*, 1989) and digested with restriction enzymes. Digested genomic DNA (10 µg) and plasmid DNA (5 pg) were separated by electrophoresis in a 1% agarose gel and transferred by capillary blotting to Hybond-N^+^ membrane. The PCR products from pTP-B and pU-I were used as probes. The probes were labelled with digoxigenin using a DIG High Prime DNA Labeling and Detection Starter kit II (Roche Applied Sciences) following the manufacturer’s instructions. Southern blot hybridization was carried out overnight after 30min of pre-hybridization at 42 °C. The hybridized probe DNA was detected by exposure to Kodak double-emulsion films at room temperature for several hours.

### Statistical analysis

Data were analysed using the Microsoft Excel and Statistical Package for the Social Sciences (Chicago, IL, USA). Statistical differences in gene expression or carotenoid content were compared using Student’s *t*-test.

## Results

### Generation of transgenic wheat lines

To explore the utilization of bacterial *CrtB* and *CrtI* genes in improving carotenoid content in wheat grains, these two genes were transformed separately and in combination into the wheat cv. Bobwhite. After herbicide-selective regeneration, positive transgenic lines were screened out in the T_0_ generation by PCR amplification for the *CrtB*, *CrtI*, or *bar* genes, with seven plants containing the *CrtI* gene, five plants containing the *CrtB* gene, and three plants containing both transgenes being identified (Table 1). Several lines of wheat transformed only with the pAHC20 plasmid were generated as vector control lines (designated VC). Self-pollination of the PCR-positive transgenic plants in the following generations led to the identification of non-segregant lines for the *CrtB* and/or *CrtI* genes. Southern blotting results for the T_3_ non-segregant transgenic lines showed that these transgenic lines had multiple insertion sites, resulting in two or more bands on the blots (Supplementary Fig. S2 at *JXB* online). The different banding patterns confirmed that these lines were derived from independent transformation events.

**Table 1.** Generation of transgenic wheat plants overexpressing bacterial CrtI and/or CrtB

Transgenic line^*a*^	Constructs	Gene(s) of interest	Selectable marker gene	No. regenerated plants	No. PCR-positive plants
OEI	pU-I, pAHC20	*CrtI*	*bar*	11	7
OEB	pTP-B, pAHC20	*CrtB*	*bar*	14	5
OEIB	pTP-B, pU-I, pAHC20	*CrtB*, *CrtI*	*bar*	11	3
VC	pAHC20	–	*bar*	28	11

^***a***^ VC is a wheat line transformed with plasmid pAHC20, which is used as transgenic control line; OEI is a line expressing *CrtI*; OEB is a line expressing *CrtB*, and OEIB has co-expression of *CrtI* and *CrtB*.

### Expression of *CrtI* and/or *CrtB* changes the seed colour of transgenic wheat

**Fig. 3.**
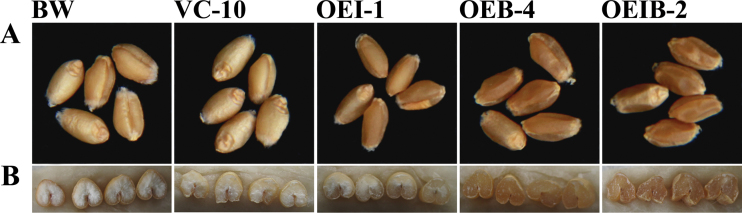
Grains phenotypes for transgenic and control wheat lines. (A) Photographs of grains from transgenic and control lines of wheat. Bobwhite (BW) is a wild-type wheat cultivar; VC-10 is a control line transformed with vector pAHC20; and OEI-1, OEB-4, and OEIB-2 are transgenic lines expressing *CrtI* or *CrtB* and co-expressing both genes, respectively. (B) Photographs of endosperms from the transgenic and control lines of wheat.

Differences in grain colour were observed among the transgenic lines and control lines. The transgenic control line VC-10 showed a similar light-yellow kernel appearance in comparison with the wild type (cv. Bobwhite), whereas three lines expressing *CrtB* and/or *CrtI* exhibited a darker red/yellow grain colour (Fig. 3A). Seeds from the OEIB-2 transgenic line, co-expressing both genes, exhibited the darkest red/yellow colour, followed by seeds from the OEB-4 line expressing *CrtB*, with the OEI-1 seeds expressing *CrtI* showing a slightly yellower colour. Moreover, the grain appearance of the three transgenic lines was slightly more wrinkled compared with that of the wild-type grains. This wrinkled kernel appearance may be related to the slight reduction in 100-grain weight, but no difference was observed in seed number per spike among the transgenic and control lines (Table 2). Unlike the distinction in kernel appearance, transverse section images revealed that the inner endosperm for line OEI-1 had a similar white colour to the control lines VC-10 and Bobwhite, and only the outer layers of seeds showed a yellower colour compared with the control lines. The endosperms from the *CrtB*-expressing line (OEB-4) and *CrtI*+*CrtB*-co-expressing line (OEIB-2) were darker red/yellow than those of the OEI-1 and control lines (Fig. 3B). The results of the grain coat and endosperm colours clearly reflected the differences in carotenoid content and composition among the transgenic and control lines.

**Table 2.** Transgene expression level, carotenoid content, 100-grain weight, and number of seeds per spike in T_3_ transgenic lines

Transgenic line (T_3_ generation)	Relative transcript levels^*a*^		Total carotenoids (μg g^–1^ of dry seed weight)^*b*^	Provitamin A (μg g^–1^ of dry seed weight)^*b*^	100-Grain weight (g)	No. seeds per spike
	***crtB***	***crtI***				
BW^c^	0	0	0.58±0.02	0.05±0.01	4.01±0.02	36.00±1.53
OIE-1	0	0.027±0.002	0.65±0.02	0.18±0.01	3.84±0.12	33.67±1.76
OEB-4	0.078±0.003	0	2.32±0.08	1.37±0.05	3.89±0.09	34.33±2.33
OEIB-2	0.091±0.004	0.037±0.002	4.06±0.49	3.21±0.37	3.96±0.06	35.33±0.88

The data represent mean values±standard error of the mean and are derived from at least three independent plants per line.

^***a***^ Relative transcript levels (normalized with respect to the β-actin transcript) were determined by qPCR.

^***b***^ Total carotenoids (sum of all carotenoid content measured) and provitamin A content (sum of α-carotene, β-cryptoxanthin, β-carotene,) were determined by HPLC analysis.

^***c***^ Bobwhite (BW) is shown as a control.

### Expression of *CrtI* and/or *CrtB* enhances carotenoid accumulation in seeds from transgenic wheat

The carotenoid composition of the transgenic and control lines in the T_2_ generation was estimated by HPLC without replication due to the seed number limitation. There was no distinction in the carotenoid composition between the Bobwhite and VC-10 control lines. The carotenoid profiles of the mature seeds from three T_2_ transgenic lines were different from that of the wild type. Several novel carotenes were detected in the transgenic wheat lines including lycopene, β-cryptoxanthin, α-carotene, and phytoene (Supplementary Table S2 at *JXB* online).

**Fig. 4.**
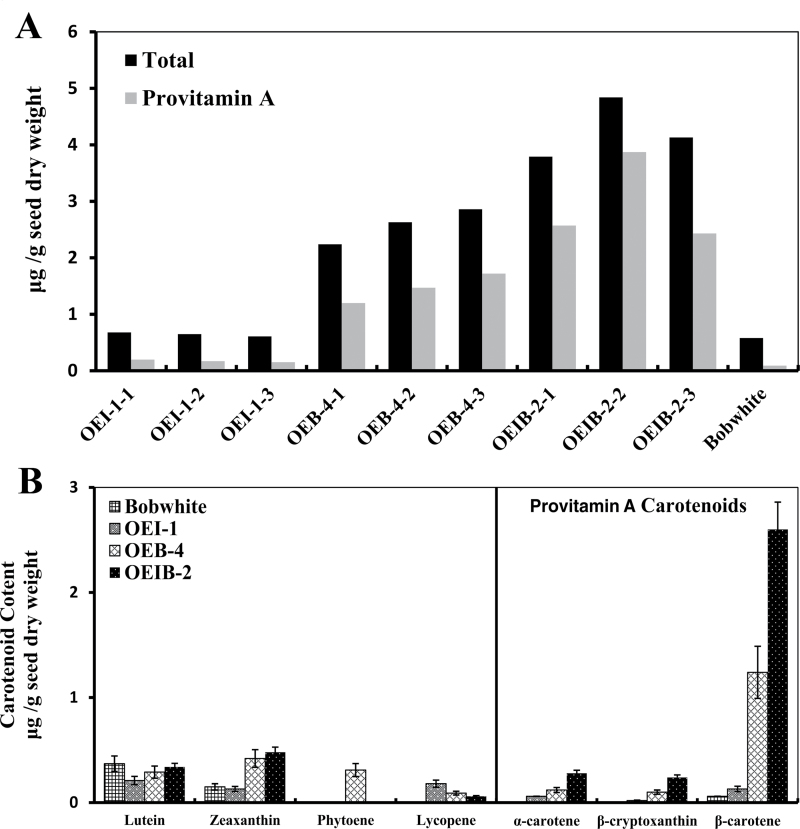
Enhancement of both total carotenoid and provitamin A content in grains from transgenic wheat with expression of *CrtI* and/or *CrtB*. (A) Total carotenoid and provitamin A content in wheat grains, harvested from three single plants from each of the T_3_ transgenic lines (OEI-1, OEB-4, and OEIB-2) and their untransformed control Bobwhite. HPLC analysis was conducted on 30 T_3_ grains harvested from each wheat line. The amount of total carotenoid is equal to the sum of all carotenoids detected, and the provitamin A content was calculated as the sum of α-carotene, β-cryptoxanthin, and β-carotene content present in the extracted samples. (B) Carotenoid composition in wheat grains the from transgenic and control lines in the T_3_ generation. The average of each carotenoid species was determined from five individual plants ears per line. Data are presented as means±standard error of the mean.

In the T_3_ generation, detailed HPLC analysis revealed significant differences in carotenoid content and composition in the seeds from the transgenic and control lines, suggesting profound changes in the carotenoid biosynthetic pathways in the transgenic lines. The total carotenoid and provitamin A content in three T_3_ plants from each of the non-segregant lines were improved (Fig. 4A). Interestingly, expression of *CrtI* did not cause difference in total carotenoid content between controls lines and the three OEI-1 plants. However, the total carotenoid content increased to 2.46 μg g^–1^ of seed dry weight in the OEB-4 plants and to 4.96 μg g^–1^ of seed dry weight in the OEIB-2 plants in contrast to the wild type. In contrast, the provitamin A content was significantly increased in all three transgenic wheat lines, ranging from 0.18 μg g^–1^ (OEI-1–3) to 3.86 μg g^–1^ of seed dry weight (OEIB-2-2). According to the differences in content of total carotenoid and provitamin A content among the plants within each line, the provitamin A content for the OEI-1, OEB-4, and OEIB-2 lines was dramatically elevated by 3.8-fold, 30-fold, and 60-fold, respectively. This result suggested that expression of the transgenes may induce an additional carotenoid substrate into the provitamin A pool in the transgenic wheat lines (26–80% of the total carotenoid pool is provitamin A in transgenic lines versus 9% in Bobwhite).

**Fig. 5.**
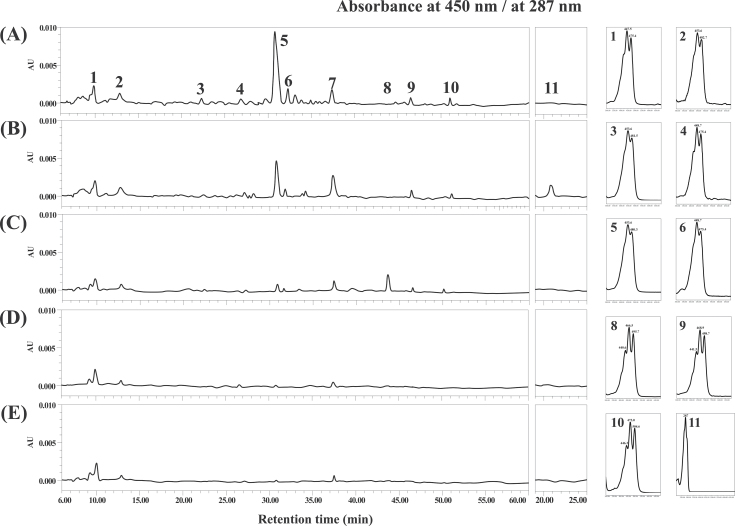
HPLC chromatograms of carotenoids extracted from grains of T_3_ transgenic and control wheat. (A) OEIB-2; (B) OEB-4; (C) OEI-1; (D) VC-10 (transgenic vector control); (E) Bobwhite (wild type). Peak 1, lutein; peak 2, zeaxanthin; peak 3, β-crptoxanthin; peak 4, α-carotene; peak 5, *trans*-β-carotene; peak 6, 9-*cis*-β-carotene; peak 7, undefined carotene; peak 8,9, *cis*-lycopene; peak 10, *trans*-lycopene; peak 11, phytoene.

The average carotenoid content of the transgenic lines OEI-1, OEB-4, and OEIB-2 for each of the major carotenoid species is shown in Fig. 4B. The HPLC results exhibited a significant increase in provitamin A carotenoids in transgenic lines, while expression of *CrtB* and/or *CrtI* had differential effects on the other carotenoid species (phytoene, lycopene, zeaxanthin, and lutein), with lutein content being slightly decreased among the transgenic and control lines (Fig. 4B). In untransformed Bobwhite, lutein was detected to be the major carotenoid species, whereas the provitamin A carotenoid species, by contrast, comprised a negligible proportion of the total carotenoid content, suggesting a bias towards the β,ε-branch in the carotenoid metabolic flux of wheat. Notably, the β-carotene levels of the OEI-1, OEB-4, and OEIB-2 lines increased up to about 3.5-fold, 27-fold, and 56-fold, respectively, in comparison with the wild type. Unlike β-carotene, other provitamin A carotenes were moderately increased in transgenic lines. Some geometric isomers of carotenoid were found in the three transgenic lines (Fig. 5). β-Carotene isomers were observed, among which 9-*cis*-β-carotene was the major component. In addition, *trans*-lycopene and *cis*-lycopene were also detected in the three transgenic lines (Supplementary Fig. S3 at *JXB* online). Thus, the significantly increased provitamin A content in the transgenic lines was due mainly to the enhancement of β-carotene levels.

The *CrtI* gene was driven by the constitutive 35S promoter, and we analysed the carotenoid content and composition in leaves of the transgenic and control lines. Due to non-expression of the *CrtB* gene in the leaf tissues of the transgenic line OEB-4, the profiles and contents of carotenoids for OEB-4 were similar to those of the control lines Bobwhite (Supplementary Fig. S4A at *JXB* online). However, lines OEI-1 and OEIB-2 showed a similar composition for carotenoids and chlorophylls, with lutein content being significantly reduced. Apart from the lutein reduction in *CrtI*-expressing lines, the content of chlorophylls and other carotenoids was not significantly changed (Supplementary Fig. S4B available at *JXB* online).

To determine whether the alteration in carotenoid composition of transgenic lines could be inherited by the following generations, we self-pollinated the T_3_ generation. In the resulting T_4_ seeds, the carotenoid content and composition were almost identical for all transgenic lines compared with the parental generation. In addition, our group has reported transgenic lines of wheat with co-expression of maize *PSY1* and bacterial *CrtI* (designated OEIP; Cong *et al.*, 2009). We therefore took advantage of the OEIP line to make a brief comparison of carotenoid composition between the previous line and lines shown in the present study. In comparison with the donor cultivar of OEIP line EM12, total carotenoid content for the OEIP line increased by about 2.6-fold, provitamin A content by 15-fold, and β-carotene by 13.5-fold (Table S3 at *JXB* online). Because the bacterial CrtB plays a role similar to maize PSY1 in the carotenoid biosynthetic pathway, we expected a similar carotenoid composition between the OEIP line and OEIB line. However, distinct from the OEIB line, several intermediate carotenoid species, including β-cryptoxanthin and lycopene, were unable to be detected with HPLC analysis (Supplementary Fig. S3 available at *JXB* online). Although these two transgenic lines (OEIP, and OEIB in the present study) have different donor wheat cultivars, Bobwhite and EM12, they exhibited a similar carotenoid composition, with the carotenoid content of EM12 being higher than that of Bobwhite. Taken into consideration the varied carotenoid composition of the two transgenic lines, we concluded that co-expression of *CrtB* and *CrtI* induces major effects on carotenoid biosynthesis in wheat endosperm.

### Elevated provitamin A content in transgenic wheat is due to increased expression levels of endogenous carotenoid biosynthetic genes

**Fig. 6.**
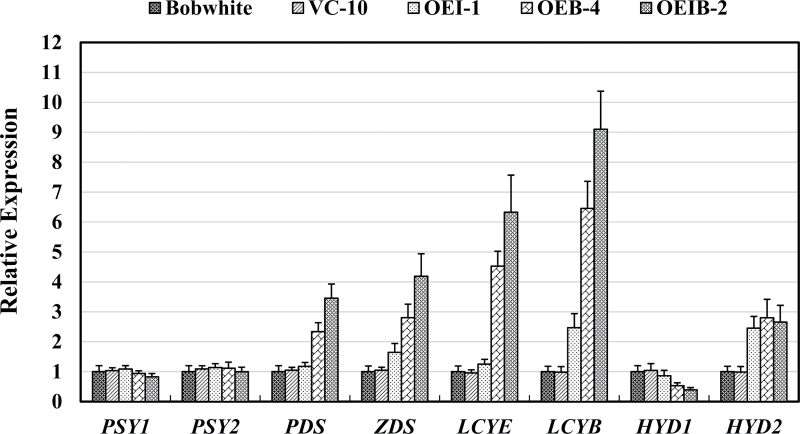
Expression levels of the endogenous carotenoid biosynthetic genes in endosperms from the transgenic and control wheat lines. Gene expression levels were measured by qRT-PCR and are determined relative to the transcript levels of the constitutively expressed β-actin gene in the same samples. Expression levels of these genes for the transformed lines are given as expression levels relative to the values for the non-transformed control line Bobwhite. qPCR results for each gene were performed for three biological replicates with three technical repeats each, and all data are shown as means±standard error of the mean.

Expression of both transgenes, *CrtB* and *CrtI*, was determined by qPCR in the endosperms of T_3_ transgenic and control wheat lines. As shown in Table 2, expression levels of *CrtI* and *CrtB* in OEIB-2 were slightly higher than the corresponding transgenes in the OEI-1 line or the OEB-4 line. Additionally, transcript levels of *CrtB* in both OEB-4 and OEIB-2 were clearly higher than those of *CrtI* in OEI-1 and OEIB-2, which concurred with the higher expression level driven by the *1Dx5* promoter than the 35S constitutive promoter. To understand further the influence of expression of *CrtI* and/or *CrtB* on endogenous carotenoid biosynthetic genes expression and on carotenoid accumulation in wheat, expression of the endogenous carotenogenic genes was also analysed in both the endosperms and leaves from transgenic and control wheat lines. In endosperm, transgenic control line VC-10 and wild-type Bobwhite showed identical expression levels for all endogenous carotenoid biosynthetic genes. In the transgenic line OEI-1, the endogenous *ZDS* gene was slightly increased, with downstream genes *LCYB* and *HYD2* being upregulated (Fig. 6). Interestingly, the expression of most carotenoid biosynthetic genes was upregulated in the transgenic lines OEB-4 and OEIB-2, with the exception of *HYD1*, which was downregulated in these two lines. In addition, expression of *PSY1* and *PSY2* was unaffected in all transgenic lines. Taken together, moderate upregulation of *LCYB* and *HYD2* in OEI-1 possibly explained its limited improvement in carotenoid content in the endosperm, while the significantly increased expression of carotenogenic genes (including *PDS*, *ZDS*, *LCYE*, *LCYB*, and *HYD2*) in the transgenic lines OEB-4 and OEIB-2 was consistent with the drastic elevation of provitamin A content.

In leaves, the transcript levels of carotenogenic genes for OEB-4 were similar to those for the untransformed control, which was consistent with the non-expression of *CrtB* (Supplementary Fig. S4C,D available at *JXB* online). However, in the leaves of OEI-1 and OEIB-2, which constitutively express *CrtI*, upregulation of *LCYB* and *HYD2* in parallel with slight repression of *PSY1*, *PDS*, *LCYE*, and *HYD1* was observed. The transcript levels of *PSY2* and *ZDS* remained stable (Supplementary Fig. S4D available at *JXB* online). This result demonstrated that constitutive expression of *CrtI* partially interfered with the carotenogenesis in leaves, which is in accordance with the decreased lutein content in these lines.

## Discussion

The nutritional value and healthy benefits conferred by carotenoids have led to increasing numbers of studies on the generation of transgenic crops with enhanced carotenoids, as exemplified in rice, potato, and maize (Paine *et al.*, 2005; Ducreux *et al.*, 2005; Aluru *et al.*, 2008). Here, the wheat cultivar Bobwhite was transformed with two carotenoid biosynthetic genes from bacteria, *CrtB* and *CrtI*, and the transgenic wheat showed significantly elevated provitamin A content. Because constitutive expression of *PSY* leads to plant dwarfism (Fray *et al.*, 1995) and the bacterial *CrtB* gene has a similar function to the plant *PSY* gene, we employed an endosperm-specific *1Dx5* promoter to drive its expression. It is worth noting that the stronger promoting activity of the *1Dx5* promoter resulted in significantly higher transcript levels of *CrtB* than that of *CrtI* in all transgenic lines, with no difference in transformation efficiencies being observed. In comparison with the *CrtI*-expressing line, higher accumulation of carotenoid content in the *CrtB*-expressing lines supports the suggestion that a tissue-specific promoter would be a better strategy for carotenoid metabolism engineering in wheat, not only for its spatio-temporal specificity of expression but also for its stronger promoting activity.

### Expression of *CrtI* in the endosperm and leaf leads to alteration of carotenoid composition

Although constitutively expressing the bacterial carotene desaturase *CrtI* in endosperm did not increase the total carotenoid content, the HPLC results demonstrated that CrtI was able to alter the profile of carotenoids in the endosperm from line OEI. Compared with the control plant, lutein was reduced in the endosperm, with β-carotene and lycopene being increased. Distinctive from the colourless rice endosperm with the expression of *CrtI* (Schaub *et al.*, 2005), the aleurone and/or the outer layers of endosperm from the OEI-1 line changed to a deeper red/yellow compared with the control lines. It is probable that the increased β-carotene and lycopene were located in the outer layer of the wheat endosperm. Additionally, besides the increased lycopene content as an expected result of the *CrtI* expression, increased β-carotene content in the OEI-1 line indicated additional endogenous lycopene β-cyclase activity (Fig. 4). Indeed, the increased transcript levels of endogenous *LCYB* helped to explain this inference (Fig. 6).

Constitutive expression of *CrtI* in the leaves of transgenic wheat lines OEI and OEIB was consistent with results from transgenic tobacco and Arabidopsis, resulting in a significant decrease in lutein content in parallel with a slight reduction in chlorophyll content (Misawa *et al.*, 1993; Galzerano *et al.*, 2014). It has been documented that the accumulation of phytoene leads to the metabolic feedback regulation of PSY and its upstream geranylgeranyl pyrophosphate synthase in the carotenoid biosynthetic pathway (Cazzonelli and Pogson, 2010). As the exclusive expression of *CrtI* in the present study could not provide additional phytoene synthase activities, chlorophyll synthesis was not significantly affected in our transgenic lines. qPCR data in the leaf tissues showed downregulation of several carotenogenic genes. It is conceivable that repression of the endogenous *LCYE* and *HYD1* genes could explain the reduction in lutein content, while upregulation of the *LCYB* and *HYD2* genes might be responsible for the stabilized levels of β-carotene and violaxanthin.

### Endosperm-specific expression of *CrtB* in wheat endosperm alters carotenoid composition and improves provitamin A content

In the *CrtB*-expressing lines (OEB-4 and OEIB-2), significantly improved total carotenoid and provitamin A content were detected. This result demonstrates that CrtB can supply an additional phytoene synthase activity in wheat endosperm. Therefore, there are two possible explanations for the accumulation of phytoene in OEB-4: (i) the additional phytoene produced in the transgenic wheat could not be accessed by PDS; or (ii) although downstream phytoene desaturases (*PDS*, *ZDS*) were upregulated, they were still not active enough to catalyse the substrate flux produced by CrtB to intermediate carotenoids downstream. Enhanced carotenoid content in transgenic plants with expression of *CrtB* has been reported in canola and flax (Shewmaker *et al.*, 1999; Fujisawa *et al.*, 2008). Our results demonstrated that exclusive expression of *CrtB* in wheat endosperm could only moderately increase the total carotenoid content and alter the carotenoid composition, suggesting that upstream of the wheat carotenoid biosynthetic pathway (probably PSY) is a rate-limiting step and that solely expressing *CrtB* has an intermediate effect on removing the restriction upstream of the pathway. This result also indicates that the expression levels and enzymatic activities of these later-acting enzymes, such as PDS, ZDS, LCYs, and HYDs, also play important roles in the regulation of carotenoid accumulation in wheat endosperm.

### Co-expression of *CrtB* and *CrtI* significantly enhances provitamin A and total carotenoid content in wheat endosperm

The darkest red/yellow endosperm colour in OEIB-2 was consistent with it having the highest total carotenoid and provitamin A content among all transgenic lines. Due to the high proportion of provitamin A in the total carotenoid amount (about 80%), we can concluded that the significant increase in total carotenoid content of the OEIB-2 was mainly due to the dramatic enhanced provitamin A. Besides the high level of β-carotene, the content of lycopene, β-cryptoxanthin, zeaxanthin, and α-carotene were also significantly improved in OEIB-2, while phytoene was not detected in the seeds from the OEIB-2 line. This indicates that the *CrtI* gene could be able to desaturate all of the produced phytoene. Some isomers of carotenes such as *cis*-lycopene and 9-*cis-*β-carotene were detected. This is different from the situation in the OEB-4 line where the *CrtISO* is in charge of forming the identical isomer equilibrium of cyclic carotenes. This suggests that the resultant isomers in *CrtI*-expressing lines have various roles because the CrtI substitutes for the plant enzyme CrtISO (Isaacson *et al.*, 2004; Moise *et al.*, 2014).

The elevation of β-carotene and zeaxanthin content in the grains from OEIB-2 was associated with xanthophylls production in the β,β*-*carotene branch of the carotenoid biosynthetic pathway. The upregulation of endogenous *LCYB* was consistent with the boost of metabolic flux in the β,β*-*carotene branch in OEIB-2, suggesting that the endogenous β,β*-*carotene branch pathway is regulated mainly by lycopene β-cyclase. Our results concur with those in transgenic maize overexpressing *CrtB* and *CrtI* (Aluru *et al.*, 2008).

We compared the expression of endogenous carotenoid biosynthetic genes among the transgenic lines with expression of *CrtB* and/or *CrtI*, which constitute the main line of the carotenoid biosynthetic pathway (from phytoene to lycopene). In the OEB-4 and OEIB-2 lines, obvious upregulation of endogenous *PDS* and *ZDS* was observed. Additional phytoene synthase activities provided by the *CrtB* gene in these lines might result in increased metabolic flux in lycopene biosynthesis, and in turn may elevate the expression of the endogenous *PDS* and *ZDS* genes. In view of the above, the accumulation of phytoene or the enhancement of metabolic flux in the pathway coincided with the upregulation of genes encoding downstream enzymes. Similarly, substantial evidence suggests a metabolic feedback regulation in the carotenoid biosynthetic pathway (Cazzonelli and Pogson, 2010). In *Arabidopsis*, the accumulation of phytoene in a *pds3* mutant led to downregulation of the *ZDS* and *LCY* genes (Qin *et al.*, 2007). In our study, without the limitation of PSY, co-expression of *CrtB* and *CrtI* resulted in upregulation of downstream genes, indicating that some downstream carotenoids might modulate the signal. Thus, although the metabolic feedback has been postulated as a mechanism to regulate carotenoid pathway, the molecular nature remains unclear.

### LCYE and LCYB are the rate-limiting enzymes of provitamin A biosynthesis in wheat

In wheat cultivar Bobwhite, lutein is the major carotenoid species and accounted for about 65% of the total carotenoid, with zeaxanthin accounting for 25%, and β-carotene for only 9% (Fig. 4). Thus, lutein is suggested to largely determine the grain colour. The unbalanced proportion between lutein and zeaxanthin exhibited a preference of carotenoid metabolic flux towards the ε,β-carotene branch in the Bobwhite cultivar. Reduction of lutein (as the predominant α-carotene-derived xanthophyll) accompanied with an increase in zeaxanthin (β-carotene-derived xanthophyll) was observed in the grains of all three transgenic lines, reflecting an obvious bias towards the β,β-carotene branch in the *CrtB* and/or *CrtI* transgenic wheat endosperms. In accordance with the alterations in carotenoid composition, upregulation of *LCYB* always coincided with enhanced provitamin A content, indicating a strong correlation between LCYB activity and provitamin A accumulation. For example, the OEIB-2 line had the highest β-carotene and provitamin A content, as well as the highest fold change in expression of *LCYB*. Taken together, co-expression of *CrtB* and *CrtI* induced the upregulation of endogenous *LCYE* and *LCYB*, which coordinately switched the ε,β-carotene-branch preference over to the β,β-carotene-branch preference. Generally, the present data demonstrate that LCYE and LCYB are the rate-limiting key enzymes downstream of the carotenoid biosynthetic pathway in wheat and that LCYB plays an essential role in converting the excess upstream flux to produce adequate provitamin A species.

### Different expression patterns of HYDs suggest their distinct roles in the carotenoid biosynthetic pathway

In the transgenic lines of wheat, the downstream metabolites in the ε,β- and β,β-carotene branches, including lutein, zeaxanthin, and β-cryptoxanthin, showed varied content. It has been reported that carotene hydroxylase (*HYD1* and *HYD2*) in wheat is differentially expressed, suggesting distinct functions in the carotenoid biosynthetic pathway (Qin *et al.*, 2012). The maize orthologue of *HYD1*, *CrtRB1*, was also demonstrated to be a principal quantitative trait locus associated with β-carotene concentration in maize kernels (Yan *et al.*, 2010). Our results showed correlations in the expression of *HYD1* and *HYD2* with the varied content of downstream carotenoid species in the transgenic lines. For instance, in the OEB-4 and OEIB-2 lines, although the majority of metabolic flux was in the β,β-carotene branch with a drastic increase in β-carotene, relatively low β-cryptoxanthin levels were paralleled by repression of the endogenous *HYD1* transcripts. On the other hand, a significant boost in the β,β-carotene branch did not lead to a decrease in carotenoid content in the ε,β-carotene branch. Upregulation of endogenous *HYD2* seemed to function in maintaining the relatively stable amounts of lutein in the transgenic lines OEB-4 and OEIB-2 (Figs 4 and 6). Several previous carotenoid analyses found that lutein levels remained constant in developing wheat grains and that it is the major carotenoid in mature whole grains, with zeaxanthin being almost undetectable (Howitt *et al.*, 2009; Qin *et al.*, 2012). Taking our results together with these previous reports, it could be hypothesized that manipulation of the upstream genes in the carotenoid biosynthetic pathway and branches (*CrtB*, *CrtI*, *LCYE*, and *LCYB*) could upregulate the metabolic flux in the β,β-carotene branch and result in enhanced β-carotene, but would not largely increase the downstream metabolites. A constant lutein content and relatively low zeaxanthin content in mature wheat grains may possibly be related to their physiological functions in grain development, maturation, and germination. Additionally, it should be noted that the transcript levels of *HYD* homeologs were not detected in our study. Differential expression of *HYD* homeologs in grain developmental stages indicates their functional diversification (Qin *et al.*, 2012) and, therefore, further characterization of *HYD* homeologs in these transgenic lines is expected to facilitate our understanding of the carotenoid biosynthetic pathways and will be of significance in metabolic engineering in wheat.

In the case of metabolic engineering in wheat endosperm, the combination of *CrtB* with *CrtI* seems to be necessary for obtaining maximal provitamin A content, which suggests that several steps of the carotenoid biosynthetic pathway should be targeted simultaneously. There is the promising of achieving a higher carotenoid accumulation in wheat endosperm through the strategy of manipulating multiple carotenogenic genes, as in the ‘Golden Potato’ and in maize (Diretto *et al.*, 2007; Zhu *et al.*, 2008). Furthermore, the endogenous carotenoid biosynthetic genes showed synergistic effects on carotenogenesis with the heterologous carotenogenic genes. Our study demonstrated that the varied expression levels of the endogenous carotenoid biosynthetic genes play important roles in carotenoid accumulation and alterations in carotenoid composition in wheat endosperm. Analysis of the transgenic lines in the present study also unveiled three important regulation nodes in the carotenoid biosynthetic pathway of hexaploid wheat: (i) phytoene formation; (ii) lycopene cyclization; and (iii) carotene hydroxylation, which could then be the targets of more detailed genetic and transgenic studies.

It is well known that the successful exploitation of transgenic cereal ‘Golden Rice’, which contains sufficient provitamin A content for complete dietary requirements, required over 8 years of effort from several scientific groups (Burkhardt *et al.*, 1997; Ye *et al.*, 2000; Paine *et al.*, 2005). The introduction of the bacterial carotenoid biosynthetic genes *CrtB* and *CrtI* led to a significant enhancement of provitamin A content in wheat grains, although it is still insufficient to combat vitamin A deficiency. However, this is still important, as a small increase in carotenoid content of wheat grains would have a large impact on the basis of the huge daily consumption of wheat-based products in populations worldwide. Our attempt to enhance the provitamin A content in hexaploid wheat through the genetic engineering will not only strengthen our knowledge of carotenoid biosynthetic regulation in wheat endosperm but will also supply predictable and sustainable metabolic engineering strategies for improving wheat nutritional quality and food utilization in the future.

## Supplementary data

Supplementary data are available at *JXB* online.

Supplementary Table S1. Primer sequences used in this study.

Supplementary Table S2. Carotenoid content and composition in T_2_ seeds from transgenic and control wheat plants.

Supplementary Table S3. Carotenoid content and composition in T_4_ seeds from transgenic and control wheat plants.

Supplementary Fig. S1. Propagation of transgenic wheat and selection of non-segregant lines for the *CrtB* and/or *CrtI* genes.

Supplementary Fig. S2. Southern blot analysis of transgenic plants harbouring transgenes.

Supplementary Fig. S3. HPLC chromatograms of carotenoids extracted from grains of the OEIP transgenic line and its control wheat.

Supplementary Fig. S4. Heterologous expression of *CrtI* alters the carotenoid composition in leaf tissue of transgenic lines and induces slight changes in the expression levels of endogenous carotenoid biosynthetic genes.

Supplementary Data

## Abbreviations:

BHT: 2,6-di-*tert*-butyl-methylphenol
HPLC: high-performance liquid chromatography
qRT-PCR: quantitative reverse transcription-PCR
TP: transit peptide
VC: vector control.
