# Supplementary material for: Enrichment of provitamin A content in wheat (Triticum aestivum L.) by introduction of the bacterial carotenoid biosynthetic genes CrtB and CrtI
Source: J Exp Bot. 2014 Apr 1;65(9):2545–56. doi: 10.1093/jxb/eru138 (PMC4036513; doi:10.1093/jxb/eru138)
Supplement: Supplementary Data [file supp_65_9_2545__index.html]

Enrichment of provitamin A content in wheat (Triticum aestivum L.) by introduction of the bacterial carotenoid biosynthetic genes CrtB and CrtI — Enrichment of provitamin A content in wheat (Triticum aestivum L.) by introduction of the bacterial carotenoid biosynthetic genes CrtB and CrtI — Supplementary Data 

# Enrichment of provitamin A content in wheat (*Triticum aestivum* L.) by introduction of the bacterial carotenoid biosynthetic genes *CrtB* and *CrtI*

## Supplementary Data

Data files

**Files in this Data Supplement:**

- Supplementary Data - Supplementary Data
