# Supplementary material for: Enrichment of provitamin A content in wheat (Triticum aestivum L.) by introduction of the bacterial carotenoid biosynthetic genes CrtB and CrtI
Source: J Exp Bot. 2014 Apr 1;65(9):2545–56. doi: 10.1093/jxb/eru138 (PMC4036513; doi:10.1093/jxb/eru138)
Supplement: Supplementary Data [file supp_eru138_jexbot111609_file001.pdf]

Supporting information

Figure S1. Propagation of transgenic wheat and selection of non-segregant lines for the *CrtB* and/or *CrtI* genes. Selection of transgenic plants in T_0 generation by PCR led to seven T_0 plants containing *CrtI* gene (designated OEI plants), five T_0 plants containing *CrtB* gene (designated OEB plants), and three T_0 plants containing both *CrtB* and *CrtI* (designated OEIB). Seeds from each single plant were harvested separately in the following generations. Because very few seeds were harvested from each T_0 plants, one or two T_1 seeds from each T_0 plant were planted to generate T_2 seeds. To evaluate the changes in carotenoid compositions in transgenic wheat, the T_2 seeds from the top-three T_1 plants that produced most seeds were used for analysis of the carotenoid contents and compositions by HPLC, with eight seeds from each of these T_1 plants being planted to produce T_2 plants. Then selection of non-segregant lines for transgens, *CrtB* and/or *CrtI*, by PCR in T1 and T_2 generations revealed three transgenic lines, OEI-1, OEB-4 and OEIB-2. Their seeds and leaves in T_3 generation were used for analyzing carotenoid compositions by HPLC and quantifying expression levels of carotenoid biosynthetic genes by qPCR.

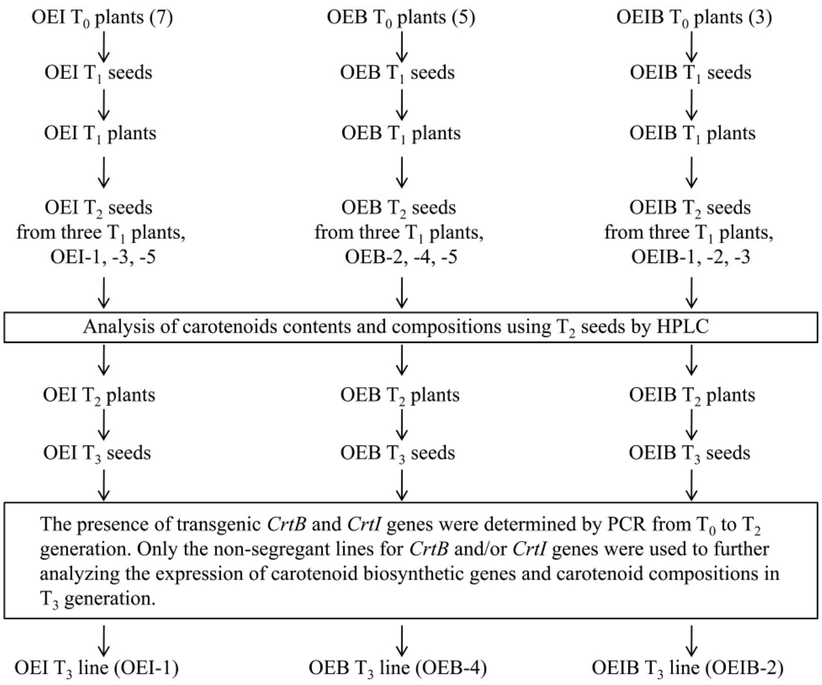

Figure S2. Southern blot analysis of transgenic plants harboring transgenes. The leaf genomic DNA digested with the single restriction enzyme *Bam*HI (*crtB*) or *Hin*dIII (*crtI*). The lines named OEI-1, OEB-4 and OEIB-2 represent independent T_3 lines regenerated with transformants. Lane -, non-transformed control (cv.Bobwhite); Lane +, positive control (pU-I or pTP-B)

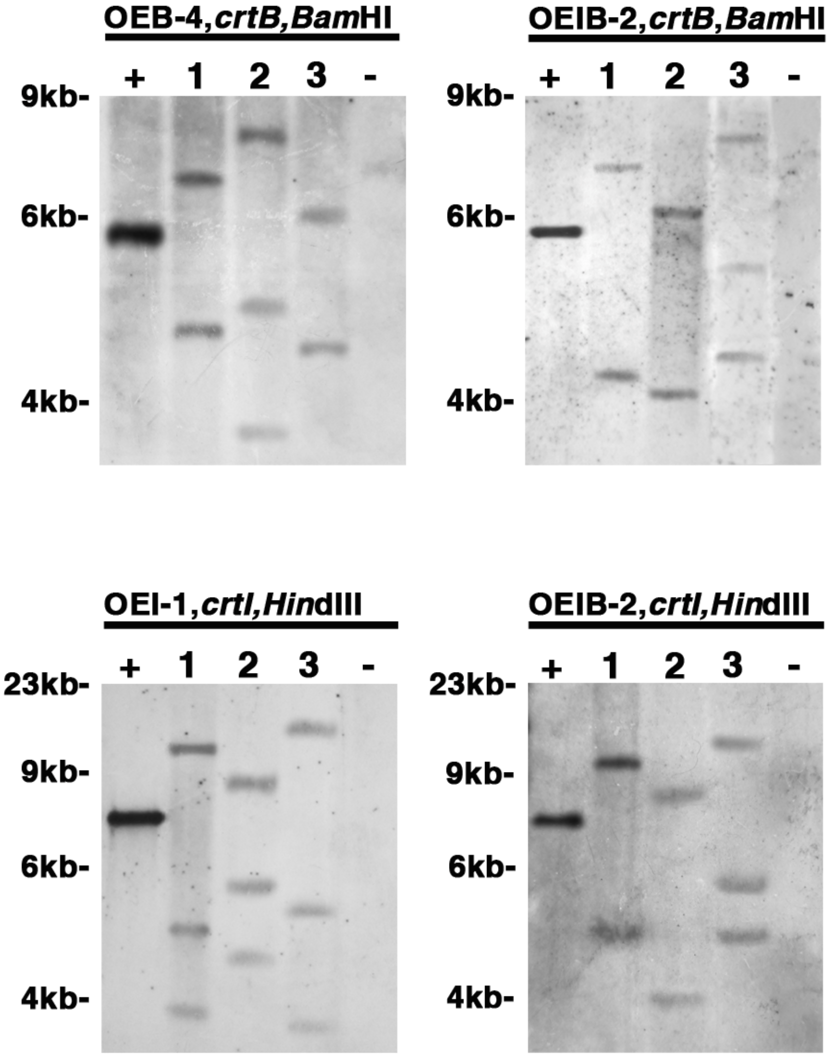

Figure S3. HPLC chromatograms of carotenoids extracted from grains of OEIP transgenic and its control wheat. (A) OEIP is line with coexpression of *CrtI* and *Mpsy*; (B) EM12 is the acceptor material of OEIP; Peak 1, lutein; Peak 2, zeaxanthin; Peak 3, *α*-carotene; Peak 4, *trans*-*β*-carotene; Peak 5, 9-*cis*-*β*-carotene;

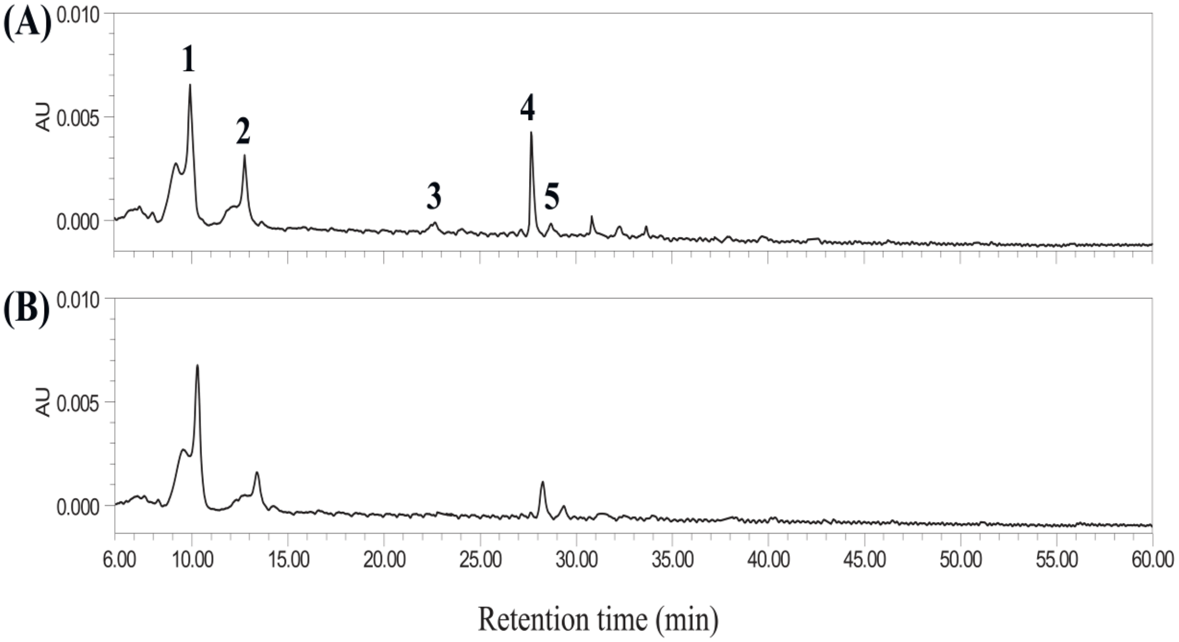

Figure S4. Heterologous expression of *CrtI* altered the carotenoid compositions in leaves of the transgenic lines and induced slight changes in the transcript levels of endogenous carotenoid biosynthetic genes. (A) HPLC chromatograms of carotenoids extracted from leaves of transgenic and its control wheat lines (OEIB-2, OEB-4, OEI-1 and BW), Peak 1, neoxanthin; Peak 2, violaxanthin; Peak 3, chlorophyll *b*; Peak 4, lutein; Peak 5, chlorophyll *a*; Peak 6, *trans*-*β*-carotene; (B) Cartenoid contents and compositions in leaves of transgenic and its control wheat lines; (C) *CrtI* Relative transcripts in leaves of transgenic and its control wheat lines; (D) Expression levels of the endogenous carotenoid biosynthetic genes in leaves from transgenic and control wheat lines. (E) Absorption spectra of carotenoids. Peak 1: neoxanthin; peak 2: violaxanthin; peak 3: Chlorophyll *b*; peak 4: lutein; peak 5: Chlorophyll *a*; Peak 6: *trans*-*β*-carotene. The data represent mean values ± SEM and are derived from leaves of four independent plants per line.

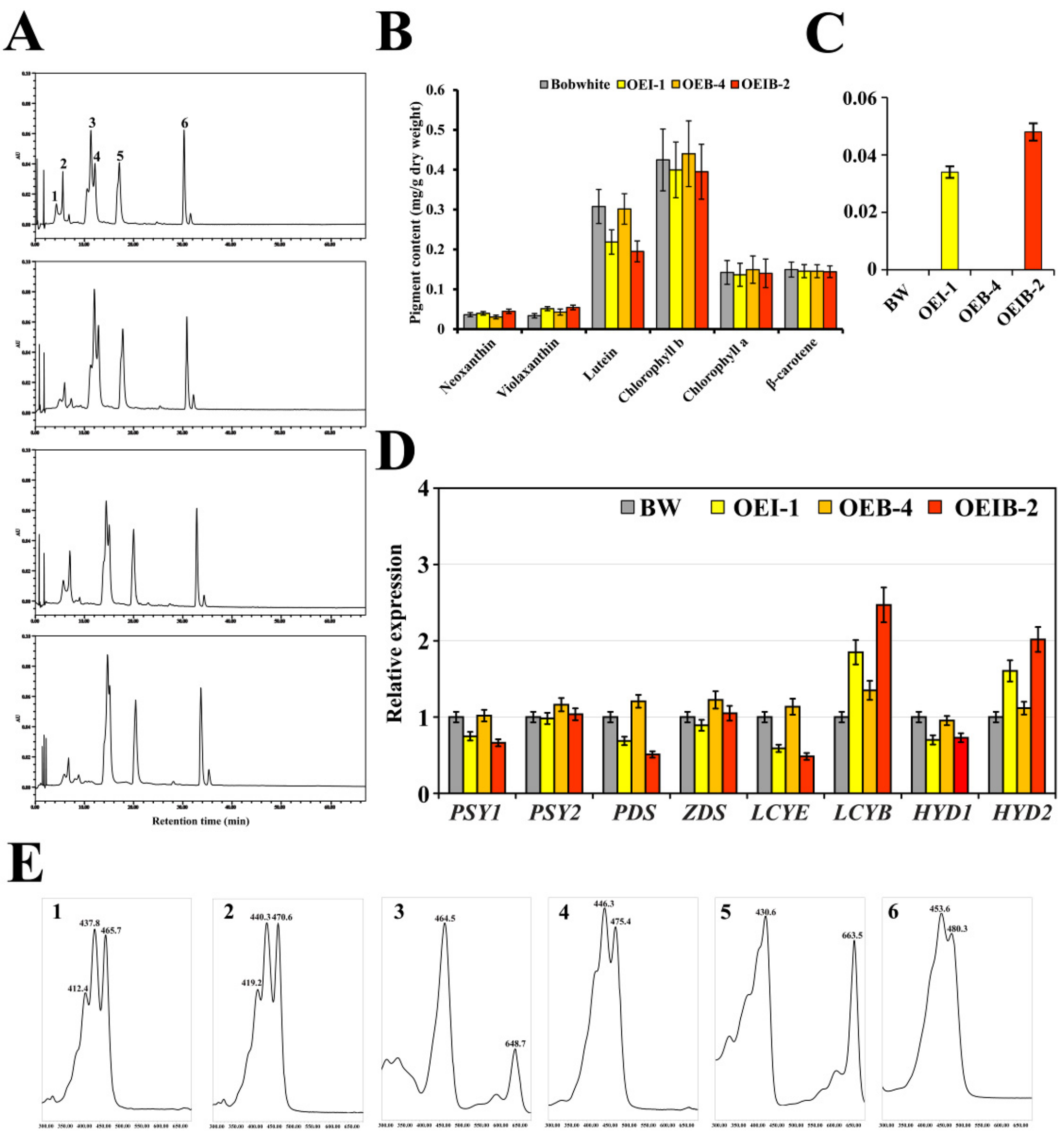

Table S1. Primer sequences used in this study

Primers name	Gene accession No.	Primer sequences (5'-to-3' direction)	Application
CrtB-F1	D90087	GTGGAAGAGTAAAGTGCATGGCAGTTGGCT	Vector construction
CrtB-R1	D90087	CTAGAGCGGGCGCTGCCAGAGA	Vector construction
TP-F1	X04334.1	ATGGCTTCTATGATATCCTCTTCCGCTGT	Vector construction
TP-R1	X04334.1	AGCCAACTGCCATGCACTTTACTCTTCCAC	Vector construction
SmaI-TP-F2	X04334.1	CCCCCGGGATGGCTTCTATGATATCCTCTTCCGCTGT	Vector construction
PstI-CrtB-R2	D90087	AAACTGCAGCTAGAGCGGGCGCTGCCAGA	Vector construction
Bar-F	X05822.1	GTCTGCACCATCGTCAACC	PCR confirmation of wheat transformants
Bar-R	X05822.1	GAAGTCCAGCTGCCAGAAAC	PCR confirmation of wheat transformants
CrtI-F1	D90087	CCCCGTCTTACTGCTTGA	PCR confirmation of wheat transformants
CrtI-R1	D90087	TTTCGCCAGTTGAGGTGC	PCR confirmation of wheat transformants
CrtB-F2	D90087	CAGCCAACTGGATGATACGC	PCR confirmation of wheat transformants
CrtB-R2	D90087	TAGCGATTGCCCAGGCGGAACG	PCR confirmation of wheat transformants
CrtB-F3	D90087	AAAGTTTTGCGACAGCCTCA	Hybridization probe
CrtB-R3	D90087	ACCAAACGACGGGCGATA	Hybridization probe
CrtI-F2	D90087	CCCCGTCTTACTGCTTGA	Hybridization probe
CrtI-R2	D90087	CCAACAGCGAGTGGAAAG	Hybridization probe
Actin-F	AB181991	AGTGGAGGTTCTACCATGTTTCCT	Quantitative PCR analysis
Actin-R	AB181991	CACTGTATTTCCTTTCAGGTGGTG	Quantitative PCR analysis
PSY1-F	EF600063	GTTTGGGCCTCTCTGTTGTTG	Quantitative PCR analysis
PSY1-R	EF600063	GCCCTCTTGGTGAAGTTGTTG	Quantitative PCR analysis
PSY2-F	GR304818.1	AAAGTGACGGATAAATGGA	Quantitative PCR analysis
PSY2-R	GR304818.1	CGCTTGGTGAAGTTGTTG	Quantitative PCR analysis
PDS-F	FJ517553	TGAACGCCCCAGTAAACCA	Quantitative PCR analysis
PDS-R	FJ517553	TTTCCGCCCAACACATCTC	Quantitative PCR analysis

ZDS-F	FJ169496	TTTAGACCTGACCAGAAGACACCA	Quantitative PCR analysis
ZDS-R	FJ169496	AATAACTCCTCTCCAGCACCACA	Quantitative PCR analysis
LCYB-F	JN622196	CGACGGTTCTTCAACGCATTCTT	Quantitative PCR analysis
LCYB-R	JN622196	TCCTGTATCAAGTTGCCGACCAT	Quantitative PCR analysis
LCYE-F	EU649786	ACACACCCTGAGGAAGCCAA	Quantitative PCR analysis
LCYE-R	EU649786	CGCATCCAACCGAGACATCAAC	Quantitative PCR analysis
CrtI-F	D90087	CGAAGGTTATCGTCAGTTTCTGG	Quantitative PCR analysis
CrtI-R	D90087	GCAGTTTCGCCAGTTGAGG	Quantitative PCR analysis
CrtB-F	D90087	CTGATGCTCTACGCCTGGTG	Quantitative PCR analysis
CrtB-R	D90087	CGTTGTTCGGGCGTTTG	Quantitative PCR analysis
HYD1-F	JX171671	ACCACATGGACAAGTTCGAGG	Quantitative PCR analysis
HYD1-R	JX171671	TTGATCCTGGCGAGCTCCT	Quantitative PCR analysis
HYD2-F	JX171673	AAGTTCGACAGCGTGCCATAC	Quantitative PCR analysis
HYD2-R	JX171673	TCCTGATCCTCCTCTGCACCT	Quantitative PCR analysis

Table S2. Cartenoid content and composition in T_2 seeds from transgenic and control wheat plants[a]

Lines [b] (T_2)	Lutein ($\mu g\ g^{-1}$)	Zeaxanthin ($\mu g\ g^{-1}$)	*β*-cryptoxanthin ($\mu g\ g^{-1}$)	Lycopene ($\mu g\ g^{-1}$)	*α*-carotene ($\mu g\ g^{-1}$)	*Trans-β*-carotene ($\mu g\ g^{-1}$)	Phytoene ($\mu g\ g^{-1}$)	Total ($\mu g\ g^{-1}$)
Bobwhite	0.38(64%)[c]	0.16(27%)	ND [d]	ND	TR[d]	0.05(9%)	ND	0.59
VC-10	0.35(69%)	0.12(24%)	ND	ND	TR	0.04(7%)	ND	0.51
OEI-1	0.20(30%)	0.13(20%)	0.02(3%)	0.14(21%)	0.02 (3%)	0.14(23%)	ND	0.65
OEI-3	0.23(35%)	0.14(21%)	0.02(3%)	0.12(18%)	0.02(3%)	0.12(20%)	ND	0.65
OEI-5	0.22(34%)	0.12(19%)	0.03(5%)	0.13(20%)	0.02(3%)	0.12(19%)	ND	0.64
OEB-2	0.29(13%)	0.41(18%)	0.09(4%)	0.04(2%)	0.11(5%)	1.08(47%)	0.29(11%)	2.31
OEB-4	0.30(11%)	0.45(18%)	0.12(4%)	0.05(2%)	0.14(5%)	1.43(51%)	0.31(11%)	2.80
OEB-5	0.28(11%)	0.38(15%)	0.10(4%)	0.04(2%)	0.12(5%)	1.22(49%)	0.34(14%)	2.48
OEIB-1	0.29(9%)	0.38(12%)	0.18(6%)	0.03(1%)	0.19(6%)	2.14(66%)	ND	3.21
OEIB-2	0.36(8 %)	0.53(11%)	0.29(6%)	0.03(1%)	0.34(7%)	3.21(67%)	ND	4.76
OEIB-3	0.32(8%)	0.46(12%)	0.24(6%)	0.03(1%)	0.25(6%)	2.47(67%)	ND	3.77

[a] Due to seed limitation in T_2 generation, carotenoid content and composition for the T_2 transgenic lines and the wild type were determined by HPLC without replication, primarily estimating the effects of heterologous expression of *crtI* and/or *crtB* on carotenoid accumulation in wheat kernels;

[b] Bobwhite is the untransformed wheat cultivar (wild-type); VC-10 is a wheat line transformed with plasmid pAHC20, which is used as transgenic control line; OEI-1, OEI-3 and OEI-5 are T_2 lines with expression of *CrtI*; OEB-2, OEB-4 and OEB-5 are T_2 lines with expression of *CrtB*; OEIB-2, OEIB-4 and OEIB-5 are T_2 lines with coexpression of *CrtI* and *CrtB*;

[c] Values in parentheses represent the percentages of each carotenoid composition relative to the total content;

[d] ND= not detected, and TR = trace amount.

Table S3. Cartenoid content and composition in T_4 seeds from transgenic and control wheat plants

Lines[a] (T4)	Lutein (μg g-1)	Zeaxanthin (μg g-1)	*β*-cryptoxanthin (μg g-1)	Lycopene (μg g-1)	*α*-carotene (μg g-1)	*β*-carotene (μg g-1)	Phytoene (μg g-1)	Total (μg g-1)
OEI-1	0.21±0.02(28%)[b]	0.14±0.01(19%)	0.04±0.01(5%)	0.18±0.01(24%)	0.04±0.01(5%)	0.13±0.01(19%)	ND	0.74±0.07
OEB-4	0.32±0.04(12%)	0.42±0.06(15%)	0.11±0.01(4%)	0.08±0.01(3%)	0.12±0.01(4%)	1.36±0.30(50%)	0.31±0.05(12%)	2.72±0.48
OEIB-2	0.39±0.05(9%)	0.48±0.07(11%)	0.34±0.04(8%)	0.04±0.01 (1%)	0.32±0.04(7%)	2.96±0.52(64%)	ND	4.53±0.83
Bobwhite	0.38±0.05(63%)	0.16±0.01(27%)	ND[c]	ND	TR[c]	0.06±0.01(10%)	ND	0.60±0.07
OEIP	0.55±0.08(20%)	0.43±0.06(15%)	ND	TR	0.18±0.01(6%)	1.64±0.30(59%)	ND	2.80±0.45
EM12	0.61±0.09 (57%)	0.34±0.04(32%)	ND	ND	TR	0.12±0.01(11%)	ND	1.07±0.14

[a] Bobwhite is the untransformed wheat cultivar (wild-type); OEI-1 is line with expression of *CrtI*; OEB-4 is line with expression of *CrtB*; OEIB-2 is line with coexpression of *CrtI* and *CrtB*; OEIP is a line with coexpression of *CrtI* and *Mpsy*, which is used as transgenic control line; EM12 is the untransformed wheat cultivar, which is the acceptor material of OEIP; Data represent the average carotenoid content (±SEM) of grains from five individual ears per line.

[b]Values in parentheses represent the percentages of each carotenoid composition relative to the total content;

[c]ND= not detected, and TR = trace amount.
